# The Moderating Effect of Religiosity on the Relationship Between Burnout and Job Satisfaction

**DOI:** 10.3389/fpsyg.2021.750493

**Published:** 2021-10-29

**Authors:** Yasemin Bal, Özgür Kökalan

**Affiliations:** ^1^Department of Business Administration, Yıldız Technical University, Istanbul, Turkey; ^2^Department of Business Administration, Istanbul Sabahattin Zaim University, Istanbul, Turkey

**Keywords:** intrinsic religious orientation, extrinsic religious orientation, burnout, job satisfaction, moderating analysis

## Abstract

The aim of this study is to investigate the moderating effect of employees’ religiosity on the relationship between their burnout and job satisfaction. The research was carried out on a total of 326 people working in the banking, education, and tourism sectors. According to the research results, a significant negative relationship was found between the burnout levels of the employees and their job satisfaction. The research also determined that intrinsic religious orientation reduced the negative effect between burnout and job satisfaction, while extrinsic religious orientation had no moderating effect on the negative relationship between burnout and job satisfaction.

## Introduction

Religion has an important effect on people’s behaviors and interacts with different aspects of life. At the same time, it has a significant impact on a person’s spirituality and psychology (Przepiorka and Sobol-Kwapinska, 2018), as well as personal development. Religious beliefs can also help individuals cope with stress and overcome difficulties. Furthermore, religion seems to be a supportive force in reducing mental pressures and crime, as well as increasing life satisfaction. Individuals express religiosity in various ways. Many religious people form the basis of their moral code based on the teachings in major religions, which express divine rules and behaviors that followers must adhere to. Both theoretical and empirical studies have been carried out on the reflections of religious beliefs and behaviors on the individual, and how they affect the behavior of people in the workplace. Numerous other studies have shown that religion can change one’s perspective on one’s job as a felt social identity in the workplace (Ekizler and Galifanova, 2020).

Although studies on religion have been around for many years, researching the effects of religion on workplace dynamics has been encountered very recently, especially with studies conducted specifically in Turkey. As stated below, there are many studies in the literature analyzing the relationship and moderating effect of religiosity on different variables, but there are no studies that specifically examine the moderating effect of religiosity on the relationship between burnout and job satisfaction. The main significance of this research is that it contributes to the literature by filling the gap in this field. Therefore, this study’s aim was to examine the moderating effect of employees’ religiosity types on the relationship between their burnout and job satisfaction. In the research, firstly, the theoretical foundations of the study were examined. Then, the data obtained within the scope of the study were analyzed with quantitative research methods, and the results were interpreted.

## Literature Review

### Religiosity

Religion is one of the most important factors that determine what people’s behaviors and attitudes should be in the workplace environment, and what the right attitude will be. As it is known, all religions in general have positive behaviors, principles, and teachings that facilitate a morally good and personally satisfying life for individuals and societies (Canda and Furman, 2009). At this point, it is necessary to mention the concept of religiosity, which is considered a basic and positive element of human development by psychologists such as Jung (1960), Maslow (1998) and Frankl (2014) who draw attention to the reflection of religion on human life. Literature proposes significant results about the effects of religiosity on people’s attitudes, behaviors, and moral perceptions in daily life and at work. According to Glock (1959), religiosity is an individual’s knowledge of the religion he or she believes in, the belief based on this knowledge, and the religious attitude and behavior that is revealed in accordance with this belief.

Allport and Ross (1967) determined the consequences and impacts of religious factors on stress. The authors revealed that individuals could be either intrinsically or extrinsically oriented to their religion. After the first studies by Allport (1950) and Allport and Ross (1967), many theoretical and empirical studies were carried out in the related field. Allport and Ross developed Religious Orientation Scale (ROS) with extrinsic and intrinsic subscale items; the scale became the most widely used scale for research in the related literature. The scale was conceptually based on Allport (1950) psychological theory of religious emotion. Allport and Ross (1967) emphasized the motivational difference that existed between the two types of religious orientations in terms of how people approached and perceived their religion. Intrinsic religious orientation (IRO) was characterized by an approach to religion which emphasized the importance of religion in the spirituality of individuals. On the other hand, extrinsic religious orientation (ERO) was emphasized by a more instrumental approach to religion which focused on accomplishing ulterior motives such as social standing, participation in a religious community, etc. (Vats et al., 2021: 86). IRO referred to a mature level of religious feeling that provided the main motive and guide for a person’s lifestyle, while ERO defined the issue of an immature level of belief that served as a means of convenience for self-serving purposes (Tiliopoulos et al., 2007). People with IRO perceived religion as the main factor that motivated them and provided meaning to their lives. People with IRO believed that the world and people were good, and they wanted to provide peace to the world in order to make it better. They internalized the values of the religion and used them as their guide in life (Allport, 1966: 455). In contrast, ERO helped people accomplish personal goals such as security, comfort, or sociability. In this situation, religion was not integrated to the deeper life and beliefs. This group of people participated in religious groups in order to gain approval from society, increase their well-being, and improve their social status in society (Allport, 1950: 59). People with this orientation used religion as a tool to reach their own ends. For these people, religious affiliation, behavior, and beliefs were a way of obtaining other things or achieving other goals (Kutcher et al., 2010).

Allport and Ross (1967) defined a person with ERO as one who used his or her religion for specific purposes, and a person with IRO as one who lived his or her religion according to its real beliefs. King and Crowther (2004) stated that people with IRO saw religious practices as an end in themselves. These people fulfilled religious requirements for their own beliefs and wishes. In contrast, the authors explained that people with ERO practiced religion for instrumental reasons such as achieving social or personal goals. Weaver and Agle (2002) argued that people with ERO used their religion in order to reach their goals, while people with IRO lived their religion according to its beliefs and values (Smither and Walker, 2015). According to the Social Identity Theory, joining a religious community led to the development and increase of an individual’s self-concept or beliefs about themselves. This may explain the benefits of religious participation on mental and physical health that provides self-efficacy for the individual. It can also be effective in making people more successful in their business lives, providing job satisfaction, and being more resistant to stress (Vats et al., 2021).

### The Relationships Among Religiosity, Burnout, and Job Satisfaction

The business environment must at least meet the mental needs of employees so that they can be more productive and effective as they improve their knowledge and skills. Job satisfaction occurs when the expectations of the employees from the workplace and the job are met. Management, leadership style, work environment, promotion, training and development opportunities, salary, and social prestige are some of the important factors in job satisfaction. Ostroff (1992) stated that job satisfaction was an important part of organizational performance. At the same time, organizational success was related to employee engagement in the workplace. Religion could be adopted to provide comfort in times of distress and to increase the social involvement of the person providing social support when needed.

There are many factors that affect the job satisfaction and organizational commitment of employees. Religiosity and spirituality are well-known factors that can increase job satisfaction, commitment, and performance (Mathew et al., 2019). People with a high level of religiosity mostly have positive attitudes such as responsibility and tolerance, and they are committed to their jobs and organizations (Darto et al., 2015). Religious teachings and values enhance individuals’ understandings about the meaning and importance of the job; therefore, it positively influences employees’ satisfaction and performance (Behere et al., 2013; Hassan et al., 2016). Additionally, religiously committed employees perceive their job positively and have more job satisfaction (Ghazzawi et al., 2016) which enables employees to be happier and more peaceful in their business life.

One of the most basic problems experienced by people who work under intense stress and pressure in business life is burnout. Burnout is a condition that occurs when physical and emotional exhaustion, constant fatigue, feelings of helplessness, and hopelessness are reflected in negative attitudes toward work, life, and other people (Maslach and Jackson, 1981). Burnout can lead to negative consequences at work such as low job satisfaction, demotivation, inefficacy, emotional exhaustion, depression, and inefficiency. This situation, which increases costs by becoming widespread in industrial societies, causes negative emotional experiences that can become permanent in the individual (Rodgerson, 1994). Burnout can occur as a result of emotional exhaustion, a loss of personality, and a decrease in personal achievement. It is usually observed in employees who are exposed to intense emotional demands in the workplace and have to work face to face with other people due to their job.

In order to avoid burnout, many organizations and leaders believe that a spiritually-minded workforce can have better attitudes toward work, and is better able to cope with stress, work collectively, and communicate ethically with colleagues (Kutcher et al., 2010). If a person’s religious beliefs are close to the general values of an organization, then he or she will be more committed to that organization. An organization must shape its values around the beliefs of its employees to increase productivity and job satisfaction. Managers must create a melting pot for managing and creating consistency (Ekizler and Galifanova, 2020).

This also affects the organizational culture of enterprises. Rust and Gabriels (2011) stated that organizations must encourage their employees to bring their whole being to work and to confirm the positive effects of religion on social behavior at work. Roundy (2009) stated that religion regulated the behavior of human beings through religious norms and values, and religiosity influenced the affective commitment of an employee. A person with IRO would be able to cope with factors such as stress and job difficulties at a higher level, and would be less likely to experience burnout. Religiosity could also reduce employee work stress and prevent burnout (Brien et al., 2021).

There were studies that found a relationship between religiosity and the physical and mental health of individuals. Some businesses were aware of this and provided environments that gave opportunities for their employees to pray during the day. Kutcher et al. (2010) found that people who practiced and lived their religion stated less burnout. The results of the research indicated that employees’ religious beliefs and practices were related positively to job satisfaction and organizational commitment. Employees who had IRO felt more committed to their organization. Religious values and beliefs made it easier for employees to be patient and endure the difficulties they faced at work, which could have a positive impact on employee productivity. Barhem et al. (2009) found that religious dimensions had a positive effect on employees’ abilities to cope with stress at work. Similarly, Navara and James (2005) determined in their research that people with higher scores of IRO had less stress levels. Laurencelle et al. (2002) also revealed that people with IRO had lower anxiety and depression scores, and a higher level of well-being. Farhadi et al. (2009) indicated that religious orientation had an effect on the life quality and mental health of employees. Furthermore, a positive correlation was found between strong religious commitment and life satisfaction, which in turn led to a higher level of job satisfaction (Hill and Pargament, 2003; Ghazzawi et al., 2016). In the research of Mensah et al. (2019), religiosity was found to have a positive effect on job satisfaction. Religiously committed employees were better able to cope with the stress and challenge of their jobs (Ghazzawi et al., 2016). When these factors are taken into account, it can be expected that the stress level of the employees will decrease, which in turn will decrease their risk of burnout. Based on the literature, the following hypotheses have been generated in our study.

*H_1_ = Burnout is negatively associated with job satisfaction.*

### Moderating Effect of Religiosity

It has been observed that religiosity has a mediating or moderating effect in studies conducted with variables such as burnout, job stress, job satisfaction, leadership, life satisfaction, and organizational commitment. Jamal and Badawi (1993) revealed that religiosity moderated the relationship between job stressors and job motivation, job satisfaction, and organizational commitment. Badawy and Mohamad (2015) explored the moderating effect of religious coping on the relationship between perfectionism and burnout. Ayten and Yıldız (2016) examined the relationship between religiosity, religious coping, and life satisfaction in a sample of retired people. According to the results of their study, religiosity had a positive effect on both positive religious coping and life satisfaction. Their findings also indicated that positive religious coping was a partial mediating factor on the relationship between religiosity and life satisfaction. In their study, Przepiorka and Sobol-Kwapinska (2018) focused on IRO and ERO as moderators of the relationship between time perspective and life satisfaction. The results indicated that religiosity acted as a buffer that reduced the strength of the negative relationship between negative time perspectives. The authors stated that religiosity was a solution for people to forget about their problems and have mental comfort. Vu (2020) stated that religious beliefs could strengthen the relationship between work-life balance and employee engagement.

Abdul et al. (2021) analyzed the moderating role of religiosity on the relationship between servant leadership and job satisfaction. The authors found that religiosity moderated the influences of servant leadership on employee performances, and it did not play any moderating role between job satisfaction and employee performances. Safara et al. (2020) found that religiosity did not have a moderating effect on the relationship between occupational stress and marital satisfaction of female nurses. As stated here, there are many studies with different variables related to the moderating effect of religiosity, but there are not any studies that specifically explore the moderating effect of religiosity on the relationship between burnout and job satisfaction. The main significance of our research is that it contributes to the literature by filling the gap in this field. This study investigates whether religiosity has a moderating effect on the relationship between burnout and job satisfaction. The following hypotheses have been generated.

*H_2_ = Intrinsic Religious Orientation moderates the relationship between burnout and job satisfaction, such that employees with higher levels of IRO report a weaker relationship between burnout and job satisfaction than employees with lower levels of IRO.*

*H3 = Extrinsic Religious Orientation moderates the relationship between burnout and job satisfaction, such that employees with higher levels of ERO report a weaker relationship between burnout and job satisfaction than employees with lower levels of ERO.*

## Materials and Methods

### Participants and Procedure

Data were collected from employees working in different service sectors in Turkey, such as the banking, education, and tourism sectors. These sectors were primarily targeted because they are all service sectors. The total population who work in the service sector in Turkey is about 16.1 million (Turkish Statistical Institute [Tuik], 2021). In the data collection process, the participants were first informed about the general aim of the study, and their consent was taken. The questionnaire was collected during 4 months. The questionnaire was distributed to participants via a Google form, one of the online survey platforms. Participants’ email addresses were taken in the data collection process, and the questionnaires were sent by email. In addition, based on the principles of snowball sampling (Biernacki and Waldorf, 1981), some participants shared the questionnaire link with their friends working in the same sector.

The questionnaire was distributed in three waves, separated by 1 month each, to reduce common method bias (Podsakoff et al., 2012). According to Matthews et al. (2014), at least 1 month’s time lag should be taken to avoid the biasing effects of occasional factors. The questionnaire consisted of four different parts. In the first part, there were questions regarding the demographic characteristics of participants. In the second part, the participants were asked to answer the burnout scale. In the third part, they were asked to complete a job satisfaction scale. In the last part, they were asked to answer the religiosity scale. At the end of the data collection process, 326 data were taken from the participants.

In this study, the sample included 156 women (47.9%) and 170 men (52.1%) with ages ranging from 20 to 60. The average work experience of respondents was of 11.7 years (SD 5.15). Of the respondents, 77.0% had at least a bachelor’s degree.

### Measure

The questionnaire used in this study consisted of three different scales called “Religiosity Scale,” “Job Satisfaction Scale,” and “Maslach Burnout Scale.”

The “Religious Orientation Scale (ROS)” used in the research was developed by Allport and Ross (1967) and translated to Turkish by Ok (2011). ROS has become one of the most widely used religiosity scales in the psychology of religion. The scale aims to measure two different constructs, namely “Intrinsic and Extrinsic Religious Orientation.” The scale, which has 20 items in total, consists of nine items that measure intrinsically motivated religiosity, and 11 items that measure extrinsic religiosity. Sample items include “I enjoy reading about my religion,” and “Although I am religious, I do not let it affect my daily life.” Items were rated on 5-point Likert scales, ranging from 1 (strongly disagree) to 5 (strongly agree). The Cronbach Alpha score of ROS was found as 0.802.

The “Job Satisfaction Scale (JSS)” used in the research is a 5-item scale developed by Brayfield and Rothe (1951) as 18 items, shortened by Judge et al. (1998), and translated to Turkish by Başol and Çömlekçi (2020). Sample items include “I feel fairly satisfied with my present job,” and “I find real enjoyment in my work.” Items were rated on 5-point Likert scales, ranging from 1 (strongly disagree) to 5 (strongly agree). The Cronbach Alpha score of JSS was found as 0.804.

The “Maslach Burnout Scale (BS)” was developed by Maslach and Jackson (1981) and translated to Turkish by İnce and Şahin (2016). BS entered the literature under Maslach’s name. It consists of 22 items and three subscales. Sample items include “I feel very energetic,” and “Working with people directly puts too much stress on me.” Items were rated on 5-point Likert scales, ranging from 1 (never) to 5 (always). The Cronbach Alpha score of JSS was found as 0.822.

In our analysis, gender, age, education level, marital status, and job experiences were taken as control variables.

### Results

#### Preliminary Analyses

In the first step, the Harman’s single-factor test was applied to evaluate common method variance. According to the test result, it is seen that no single factor can explain the majority of the variance. The first factor explained only 18.94% of the total variance. This result showed that there was no common method bias in this study.

In Table 1, the means, standard deviations, and correlations among variables are shown. Table 1 reports on the descriptive statistics of the data of the sample.

**TABLE 1:** Descriptive statistics.

	Mean	SD	AVE	MSV	CR	1	2	3	4
(1) Intrinsic religious orientation (IRO)	4.09	0.691	0.59	0.44	0.78	1			
(2) Extrinsic religious orientation (ERO)	2.71	0.674	0.61	0.42	0.82	−0.095	1		
(3) Job satisfaction (JS)	3.73	0.659	0.67	0.38	0.80	0.224**	−0.076	1	
(4) Burnout (BO)	2.77	0.436	0.65	0.45	0.82	−0.097	0.260**	−0.340**	1
(5) Gender	1.52	0.500				0.0.16	0.052	0.001	−0.021
(6) Marital status	1.59	0.492				0.0.46	−0.099	0.034	0.055
(7) Education	3.80	1.085				−0.126*	−0.177**	−0.044	0.083
(8) Age	35.6	8.955				0.200**	−0.031	0.172**	0.105
(9) Work experience	11.76	5.151				0.097	0.051	0.090	−0.089

N = 326; **Correlation is significant at the 0.01 level (2-tailed). *Correlation is significant at the 0.05 level (2-tailed).

The average score on the IRO and ERO were 4.09 ± 0.69 and 2.71 ± 0.67, respectively. The independent sample *t*-test of IRO and ERO scores showed that there was no statistically significant difference between male and female (*t* = −0.93, *p* = 0.35; *t* = 0.29, *p* = 0.76; *t* = 1.40). The one-way ANOVA test results showed that there were no significant differences in IRO and ERO scores according to education level (*F* = 1.63, *p* = 0.11; *F* = 1.86, *p* = 0.07).

The average score on the JS was 3.73 ± 0.65. According to the test result, JS scores showed no statistically significant difference between male and female (*t* = −0.11, *p* = 0.99). The one-way ANOVA test results showed no significant differences in JS scores according to education level (*F* = 0.55, *p* = 0.69).

The average score on the BO was 2.77 ± 0.43. According to the test result, BO scores showed no statistically significant difference between male and female (*t* = 0.37, *p* = 0.71). The one-way ANOVA test results showed no significant differences in BO scores according to education level (*F* = 2.16, *p* = 0.07).

Correlation analysis showed that IRO was positively and statistically correlated with job satisfaction (*r* = 0.224, *p* < 0.01), and ERO had a positive correlation with burnout (*r* = 0.260, *p* < 0.01). It was also seen that they were positively correlated with IRO and ERO (*r* = −0.126, *p* < 0.05; *r* = −0.177, *p* < 0.05).

#### Hypotheses Testing

Covariance-Based Structural equation modeling (CB-SEM) was used to examine the hypothesized moderation using IBM SPSS Amos version 25. CB-SEM was used in the analysis since the sample size are not small. As a preliminary step in CB-SEM, normality of the data was checked by the kurtosis and skewness of the data, because normality in the data should be provided to use CB-SEM. Kurtosis and skewness of the data fall within a range of -1.96 to 1.96. These values showed that the data are normally distributed (Meydan and şeşen, 2015). The other step, confirmatory factor analysis (CFA) was used to assess the factor structure and validate the scales. In the model fit assessment, chi-square test (χ^2^), goodness-of-fit index (GFI), adjusted goodness-of-fit index (AGFI), comparative fit index (CFI), and root-mean squared error of approximation (RMSEA). According to the cut-offs of the fit indices, GFI and CFI values larger than 0.90, AGFI value lager than 0.85, and RMSEA value less than 0.10 (Sümer, 2000; Meydan and şeşen, 2015). Although there are studies in the literature stating that fit index values should be higher, these values were found to be sufficient for acceptable fit in structural equation modeling. According to the results of CFA (x2 = 148.74; df = 37; *p* = 0.000, GFI = 0.92, AGFI = 0.87, CFI = 0.91, RMSEA = 0.098), the model had an acceptable goodness of fit.

Hypothesis 1 proposes that burnout is associated with job satisfaction. Table 2 reports that burnout is negatively associated with job satisfaction (β = −0.493, *t* = −6.25, SE = 0.08, *p* < 0.01). Hypothesis 1 is supported.

**TABLE 2:** Intrinsic religious orientation (IRO) moderating analysis.

	Job satisfaction			
	H_1_		H_2_	
	β	SE	β	SE
Constant	4.888**	0.243	4.889**	0.239
Gender	–0.062	0.042	–0.047	0.031
Marital status	0.060	0.123	0.003	0.092
Education level	0.016	0.121	0.030	0.108
Age	0.096*	0.038	0.054	0.038
Work experience	–0.039	0.183	–0.343	0.176
Burnout (BO)	−0.493**	0.081	−0.729**	0.102
Intrinsic religious orientation (IRO)			0.343*	0.076
BO × IRO			0.063**	0.018
*R* ^2^	0.13		0.16	
*F*	25.12**		21.53**	

*p < 0.05; **p < 0.01.

**FIGURE 1:**
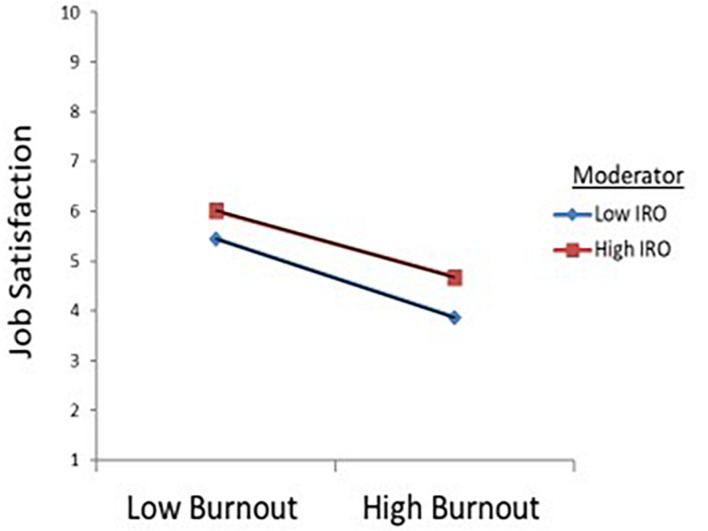
The interaction between burnout and intrinsic religious orientation (IRO).

Hypothesis 2 proposes that IRO moderates the impact of burnout on job satisfaction, such that an employee with high levels of IRO would be more likely to have more satisfaction than an employee with less IRO. Table 2 reports that the interaction term was statistically significant (β = 0.063, *t* = 9.71, SE = 0.01, *p* < 0.01). According to the analysis result, IRO dampens the negative relationship between burnout and job satisfaction. Figure 1 depicts this relationship. Hypothesis 2 is supported.

**FIGURE 2:**
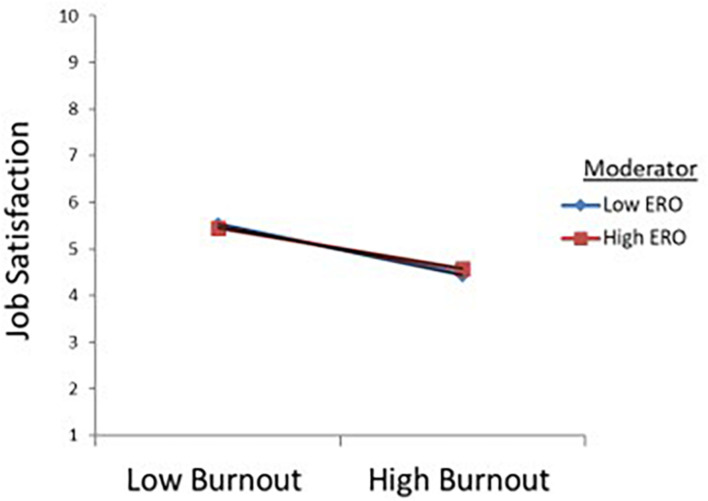
The interaction between burnout and extrinsic religious orientation (ERO).

Hypothesis 3 proposes that ERO moderates the impact of burnout on job satisfaction, such that an employee with high levels of ERO would be more likely to have more satisfaction than an employee with less ERO. Table 3 reports that the interaction term was not statistically significant (β = 0.054, *t* = 0.76, SE = 0.01, *p* > 0.05). According to the analysis result, it is seen that ERO has no moderator role relationship between burnout and job satisfaction. Figure 2 depicts this relationship. Hypothesis 3 is not supported.

**TABLE 3:** Extrinsic religious orientation (ERO) moderating analysis.

	Job satisfaction			
	H_1_		H_2_	
	β	SE	β	SE
Constant	4.888**	0.243	4.888**	0.243
Gender	–0.062	0.042	–0.062	0.042
Marital status	0.060	0.123	0.060	0.124
Education level	0.016	0.121	0.016	0.121
Age	0.096*	0.038	0.096*	0.038
Work experience	–0.039	0.183	–0.039	0.183
Burnout (BO)	−0.493**	0.081	−0.493**	0.081
Intrinsic religious orientation (IRO)			0.014	0.006
BO × IRO			0.054	0.011
*R* ^2^	0.13		0.13	
*F*	25.12**		25.12**	

*p < 0.05; **p < 0.01.

#### Robustness Check

After performing a moderating analysis, robustness checks were conducted on the data. The test results are depicted in Tables 4, 5. The results of the robustness analysis were found to be consistent with the previous analysis. Burnout has a negative effect on job satisfaction. IRO moderates the relationship between burnout and job satisfaction. However, ERO has no moderating role on the relationship between burnout and job satisfaction.

**TABLE 4:** Output of robustness analysis for IRO.

	Job satisfaction	
	H_1_	H_2_
	β	β
Constant	4.911**	4.911**
Gender	–0.062	–0.057
Marital status	0.063	0.007
Education level	0.019	0.036
Age	0.101*	0.076
Work experience	–0.072	–0.328
Burnout (BO)	−0.498**	−0.736**
Intrinsic Religious Orientation (IRO)		0.351*
BO × IRO		0.066**
*R* ^2^	0.14	0.17
*F*	28.121**	23.173**

*p < 0.05; **p < 0.01.

**TABLE 5:** Output of robustness analysis for ERO.

	Job satisfaction	
	H_1_	H_2_
	β	β
Constant	4.911**	4.912**
Gender	–0.062	–0.066
Marital status	0.063	0.062
Education level	0.019	0.023
Age	0.101*	0.102*
Work experience	–0.072	–0.051
Burnout (BO)	−0.498**	−0.489**
Intrinsic religious orientation (IRO)		0.015
BO × IRO		0.051
*R* ^2^	0.14	0.13
*F*	28.121**	25.341**

*p < 0.05; **p < 0.01.

## Discussion and Conclusion

Religious behavior affects many organizational outcomes. It is known that employees with a high level of internal religiosity can cope with stress factors more easily in the work environment. Employees who have IRO feel more committed to their organization. This allows employees to experience less burnout because religious teachings generally encourage doing the job properly. It is known that individuals with IRO have higher job-related motivations and therefore feel more job satisfaction. Various studies in the literature support that religion is associated with being happier and more satisfied at work. There are also studies examining whether religiosity has a mediating or moderating effect with different variables such as burnout, job satisfaction, job stress, leadership, life satisfaction, and organizational commitment (Jamal and Badawi, 1993; Badawy and Mohamad, 2015; Ayten and Yıldız, 2016; Przepiorka and Sobol-Kwapinska, 2018; Safara et al., 2020; Vu, 2020; Abdul et al., 2021). Although there are studies in the literature investigating the relationship between burnout and religiosity, and between job satisfaction and religiosity, there are no studies investigating the moderating effect of religiosity on the relationship between these two variables. Within the scope of the study, we examine whether religiosity has a moderating effect on the relationship between job satisfaction and burnout. The main significance of our research is that it contributes to the literature by filling the gap in the related field.

It is known that the stress and burnout levels of employees negatively affect job satisfaction. According to the analysis result, IRO dampens the negative relationship between burnout and job satisfaction. People with high levels of religiosity mostly have positive attitudes such as responsibility and tolerance toward their work (Jamal and Badawi, 1993; Navara and James, 2005; Barhem et al., 2009; Kutcher et al., 2010; Darto et al., 2015; Bednarczuk, 2019; Mensah et al., 2019; Vu, 2020). Employees who truly experience religiosity with strong internally oriented emotions have a high level of satisfaction with their work, are more committed to their institutions, and experience less burnout by coping with stress factors more easily due to the emotions that come with their religious beliefs. It is very important for managerial practices to determine the variables that may have a role in reducing the burnout level of employees and increasing their job satisfaction. Identifying the values that employees prioritize is important to create harmony between managers and employees in the organization. Determining the values that employees care about, and providing opportunities for employees to implement these priorities will be effective in reducing the stress of employees and increasing their job satisfaction. Through these values, organizations can identify gaps and needs that play an important role in outcomes such as employee performance, engagement, satisfaction, and commitment. Our study is important in terms of revealing these results. Furthermore, the study presents a different perspective in terms of giving an example from Turkey. In future studies, the moderating effect of religiosity can be investigated comparatively with samples from various sectors and different variables. As in almost every study, there are limitations in this study as well. The most important limitation of the study is the number of participants. Although a statistically sufficient number of participants have been used as a sample size, further studies with more participants may provide more reliable results.

## Data Availability Statement

The raw data supporting the conclusions of this article will be made available by the authors, without undue reservation.

## Ethics Statement

Ethical review and approval was not required for the study on human participants in accordance with the local legislation and institutional requirements. The patients/participants provided their written informed consent to participate in this study.

## Author Contributions

YB organized the theoretical background and wrote the literature review. ÖK performed the statistical analysis and wrote the first draft of the manuscript. YB and ÖK designed the study conceptually and methodically, wrote sections of the manuscript, contributed to the article, read, revised, and approved the submitted version of the manuscript.

## Conflict of Interest

The authors declare that the research was conducted in the absence of any commercial or financial relationships that could be construed as a potential conflict of interest.

## Publisher’s Note

All claims expressed in this article are solely those of the authors and do not necessarily represent those of their affiliated organizations, or those of the publisher, the editors and the reviewers. Any product that may be evaluated in this article, or claim that may be made by its manufacturer, is not guaranteed or endorsed by the publisher.
